# Cerebrospinal fluid proteomic profiling of cognitively unimpaired individuals with suspected non-Alzheimer's disease pathophysiology

**DOI:** 10.1093/braincomms/fcaf253

**Published:** 2025-06-20

**Authors:** Aurore Delvenne, Johan Gobom, Lianne M Reus, Valerija Dobricic, Mara ten Kate, Suzanne E Schindler, Inez Ramakers, Betty M Tijms, Rik Vandenberghe, Jolien Schaeverbeke, Pablo Martinez-Lage, Mikel Tainta, Charlotte E Teunissen, Julius Popp, Gwendoline Peyratout, Magda Tsolaki, Yvonne Freund-Levi, Simon Lovestone, Johannes Streffer, Frederik Barkhof, Lars Bertram, Kaj Blennow, Henrik Zetterberg, Pieter Jelle Visser, Stephanie J B Vos

**Affiliations:** Department of Psychiatry and Neuropsychology, Alzheimer Centrum Limburg, School for Mental Health and Neuroscience, Maastricht University, Maastricht 6229 ER, The Netherlands; Clinical Neurochemistry Laboratory, Sahlgrenska University Hospital, Mölndal 431 80, Sweden; Department of Psychiatry and Neurochemistry, Institute of Neuroscience and Physiology, Sahlgrenska Academy at the University of Gothenburg, Mölndal 431 41, Sweden; Department of Neurology, Alzheimer Center Amsterdam, Amsterdam Neuroscience, Vrije Universiteit Amsterdam, Amsterdam UMC, Amsterdam 1081 HZ, The Netherlands; Lübeck Interdisciplinary Platform for Genome Analytics, University of Lübeck, Lübeck 23562, Germany; Department of Neurology, Alzheimer Center Amsterdam, Amsterdam Neuroscience, Vrije Universiteit Amsterdam, Amsterdam UMC, Amsterdam 1081 HZ, The Netherlands; Department of Radiology and Nuclear Medicine, Amsterdam Neuroscience, Vrije Universiteit Amsterdam, Amsterdam UMC, Amsterdam 1081 BT, The Netherlands; Department of Neurology, Washington University School of Medicine, St. Louis, MO 63110, USA; Knight Alzheimer's Disease Research Center, Washington University School of Medicine, St. Louis, MO 63108, USA; Department of Psychiatry and Neuropsychology, Alzheimer Centrum Limburg, School for Mental Health and Neuroscience, Maastricht University, Maastricht 6229 ER, The Netherlands; Department of Neurology, Alzheimer Center Amsterdam, Amsterdam Neuroscience, Vrije Universiteit Amsterdam, Amsterdam UMC, Amsterdam 1081 HZ, The Netherlands; Neurology Service, University Hospitals Leuven, Leuven 3000, Belgium; Department of Neurosciences, Laboratory for Cognitive Neurology, KU Leuven, Leuven 3000, Belgium; Neurology Service, University Hospitals Leuven, Leuven 3000, Belgium; Department of Neurosciences, Laboratory for Cognitive Neurology, KU Leuven, Leuven 3000, Belgium; Centre for Research and Memory Clinic, Fundación CITA-Alzhéimer Fundazioa, Donostia—San Sebastián Gipuzkoa 20009, Spain; Centre for Research and Memory Clinic, Fundación CITA-Alzhéimer Fundazioa, Donostia—San Sebastián Gipuzkoa 20009, Spain; Department of Clinical Chemistry, Neurochemistry Laboratory, Amsterdam University Medical Centers (AUMC), Amsterdam Neuroscience, Amsterdam 1081 HV, Netherlands; Old Age Psychiatry, University Hospital Lausanne, Lausanne 1005, Switzerland; Department of Psychiatry, Psychotherapy and Psychosomatics, Psychiatry University Hospital Zürich, Zürich 8008, Switzerland; Old Age Psychiatry, University Hospital Lausanne, Lausanne 1005, Switzerland; 1st Department of Neurology, AHEPA University Hospital, Medical School, Faculty of Health Sciences, Aristotle University of Thessaloniki, Thessaloniki 546 36, Greece; Department of Neurobiology, Caring Sciences and Society (NVS), Division of Clinical Geriatrics, Karolinska Institutet, Stockholm 141 57, Sweden; Department of Psychiatry in Region Örebro County and School of Medical Sciences, Faculty of Medicine and Health, Örebro University, Örebro 703 62, Sweden; Department of Old Age Psychiatry, Psychology and Neuroscience, King's College, London SE5 8AB, United Kingdom; Department of Psychiatry, University of Oxford, Oxford OX1 2JD, United Kingdom; Department of Biomedical Sciences, Reference Center for Biological Markers of Dementia (BIODEM), University of Antwerp, Antwerp 2610, Belgium; H. Lundbeck A/S, Valby 2500, Denmark; Department of Radiology and Nuclear Medicine, Amsterdam Neuroscience, Vrije Universiteit Amsterdam, Amsterdam UMC, Amsterdam 1081 BT, The Netherlands; Queen Square Institute of Neurology and Centre for Medical Image Computing, University College London, London WC1N 3BG, United Kingdom; Lübeck Interdisciplinary Platform for Genome Analytics, University of Lübeck, Lübeck 23562, Germany; Clinical Neurochemistry Laboratory, Sahlgrenska University Hospital, Mölndal 431 80, Sweden; Department of Psychiatry and Neurochemistry, Institute of Neuroscience and Physiology, Sahlgrenska Academy at the University of Gothenburg, Mölndal 431 41, Sweden; Paris Brain Institute, ICM, Pitié-Salpêtrière Hospital, Sorbonne University, Paris 75013, France; Division of Life Sciences and Medicine, and Department of Neurology, Neurodegenerative Disorder Research Center, Institute on Aging and Brain Disorders, University of Science and Technology of China and First Affiliated Hospital of USTC, Hefei, Anhui 230026, PR China; Clinical Neurochemistry Laboratory, Sahlgrenska University Hospital, Mölndal 431 80, Sweden; Department of Psychiatry and Neurochemistry, Institute of Neuroscience and Physiology, Sahlgrenska Academy at the University of Gothenburg, Mölndal 431 41, Sweden; Department of Neurodegenerative Disease, UCL Institute of Neurology, London WC1N 3BG, United Kingdom; Fluid Biomarker Laboratory, UK Dementia Research Institute at UCL, London W1T 7NF, United Kingdom; Hong Kong Center for Neurodegenerative Diseases, Clear Water Bay, Hong Kong, China; Wisconsin Alzheimer’s Disease Research Center, University of Wisconsin School of Medicine and Public Health, University of Wisconsin-Madison, Madison, WI 53792, USA; Department of Psychiatry and Neuropsychology, Alzheimer Centrum Limburg, School for Mental Health and Neuroscience, Maastricht University, Maastricht 6229 ER, The Netherlands; Department of Neurology, Alzheimer Center Amsterdam, Amsterdam Neuroscience, Vrije Universiteit Amsterdam, Amsterdam UMC, Amsterdam 1081 HZ, The Netherlands; Division of Neurogeriatrics, Department of Neurobiology, Care Sciences and Society, Karolinska Institutet, Stockholm 141 57, Sweden; Department of Psychiatry and Neuropsychology, Alzheimer Centrum Limburg, School for Mental Health and Neuroscience, Maastricht University, Maastricht 6229 ER, The Netherlands

**Keywords:** suspected non-Alzheimer’s disease pathophysiology, cognitively unimpaired, biomarkers, tau, proteomics

## Abstract

Suspected non-Alzheimer's disease pathophysiology (SNAP) is a biomarker-based concept describing individuals with abnormal tau and/or neurodegeneration markers but normal amyloid levels. SNAP is common in individuals with normal cognition (NC), but its underlying pathophysiology is understudied, while being relevant for clinical trial design and treatment approaches. We aimed to investigate the pathophysiology of individuals with NC who are amyloid-negative and tau-positive (SNAP) through cerebrospinal fluid (CSF) proteomics. Two hundred and ninety-one individuals with NC were classified based on CSF amyloid β1-42 and phosphorylated tau 181, as amyloid-negative/tau-negative (controls), amyloid-negative/tau-positive (SNAP), amyloid-positive/tau-negative and amyloid-positive/tau-positive. We measured 3102 proteins in CSF using tandem mass tag proteomic analyses. We compared protein abundance between groups using analysis of covariance and identified enriched biological pathways using Gene Ontology. We also examined differences between groups in genetic risk for Alzheimer’s disease, estimated using polygenic risk scores based on genome-wide association study data. SNAP individuals with NC showed mostly increased protein levels (*n* = 360) compared with controls, mainly associated with neuroplasticity, angiogenesis, and protein modification and degradation. The proteomic profile of SNAP was similar to that of amyloid-positive/tau-positive individuals, while distinct from amyloid-positive/tau-negative individuals, who showed mainly decreased proteins associated with neuroplasticity. Higher levels of amyloid β1-40 and amyloid β1-42 were observed in SNAP compared with the three other groups. Polygenic risk scores analyses showed no significant differences between SNAP, amyloid-positive/tau-negative, and amyloid-positive/tau-positive individuals, while SNAP showed some genetic differences from controls, which were driven by *APOE*. Individuals with NC and SNAP or amyloid-positive/tau-positive status showed similar CSF proteomic profiles, while amyloid-positive/tau-negative individuals showed a distinct CSF proteomic profile. This suggests that tau, rather than amyloid, might be the main driver of the proteomic profiles in SNAP and other amyloid/tau subgroups. This may have implications for future proteomic studies and clinical trial design, as these findings highlight the importance of considering tau status in future studies.

## Teaser

Suspected non-Alzheimer’s disease pathophysiology (SNAP) refers to individuals with abnormal tau but normal amyloid. Delvenne *et al*. investigated the pathophysiology of SNAP in cognitively unimpaired individuals using cerebrospinal fluid proteomics and found that SNAP individuals exhibit increased neuroplasticity proteins, similar to those in Alzheimer's disease, suggesting that tau drives these changes.

## Graphical Abstract

**Graphical Abstract:**
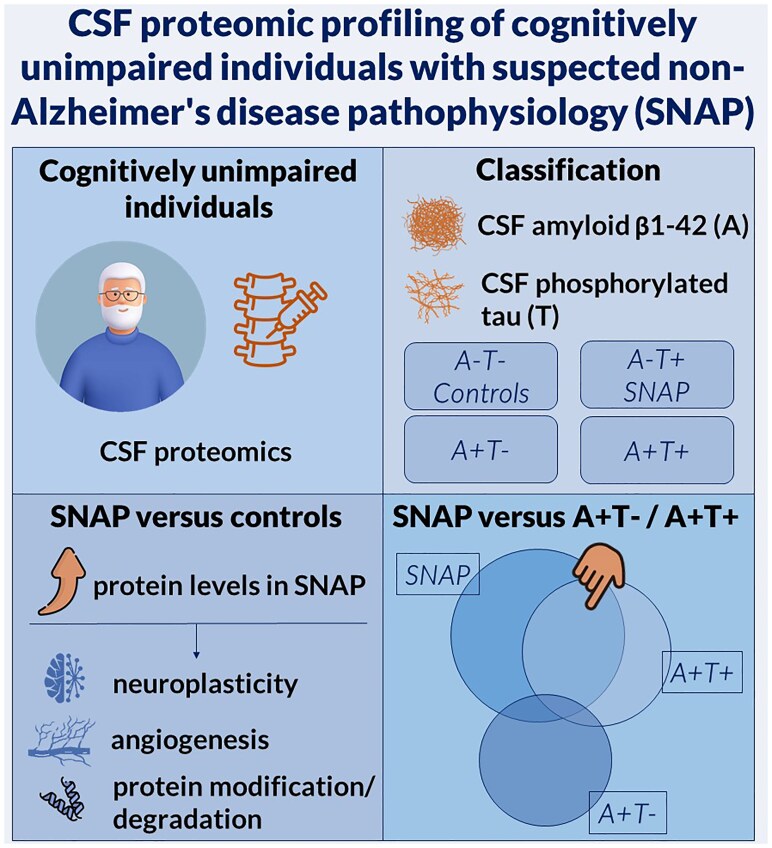

## Introduction

Suspected non-Alzheimer's disease pathophysiology (SNAP) is a biomarker-based concept referring to individuals with a normal amyloid-β protein (Aβ) marker (A−), and abnormal tau (T+) and/or neurodegeneration (N+) marker(s), defined by cerebrospinal fluid (CSF) or imaging markers.^1,2^ In the present paper, SNAP is defined as normal CSF Aβ-42 levels and abnormal CSF p-tau levels (A−T+). Globally, SNAP (A−T+ or A−N+) is common (∼20%) among individuals with normal cognition (NC).^3^ While the underlying pathophysiology of A−T+ individuals with NC is still largely unknown, this knowledge will likely have implications for trial design and treatment development for individuals with NC-SNAP.

Previous studies have demonstrated that NC-SNAP (A–T+ or A−N+) is associated with lower apolipoprotein E (*APOE*) ɛ4 frequency compared with individuals with NC and abnormal amyloid (A+), but similar to controls.^4-6^ NC A−T+ has shown a higher vascular burden than controls, but similar as NC A+T+.^6-9^ While SNAP in individuals with mild cognitive impairment (MCI) has been shown to be associated with cognitive decline, previous studies have shown that NC-SNAP A−T+ or A−N+ individuals remains relatively cognitively stable after a mean follow-up time of 7 years.^4,6,10^ Yet, higher CSF levels of APPβ and immune and inflammatory markers (TREM2, YKL-40 and CX3CL1) have been observed in NC A−T+ compared with controls and NC A+T−, but similar to NC A+T+.^11,12^ Higher levels of clusterin, which promotes Aβ clearance,^13^ have been found in NC A−T+ compared with controls, NC A+T− and NC A+T+.^12^ To date, it is still unclear whether NC-SNAP represents early Alzheimer’s disease (AD) or may reflect other non-Alzheimer’s disease underlying processes, and whether NC-SNAP has similar genetic risk for Alzheimer’s disease as persons with NC and abnormal amyloid.

In the present study, we aimed to investigate the pathophysiology of NC A−T+ (SNAP) using large-scale CSF proteomics, allowing the assessment of a substantial number of proteins at once. We compared the proteomic profile of NC A−T+ (normal CSF Aβ42 and abnormal CSF p-tau, A−T+; *n* = 30) to that of controls (NC A−T−; *n* = 141), and preclinical Alzheimer’s disease (NC A+T− (*n* = 65) and NC A+T+ (*n* = 55)). We characterized proteins with significantly different levels by Gene Ontology (GO) analysis, as well as cell type- and brain region–specific gene expression analysis. In addition, we examined differences in genetic risk for Alzheimer’s disease, as estimated using AD polygenic risk scores (PGRS), between NC A−T+, controls and preclinical Alzheimer’s disease biomarker groups.

## Materials and methods

### Participants

Two hundred and ninety-one participants were included from the Maastricht BioBank Alzheimer Centre Limburg cohort (BB-ACL, *n* = 42) memory clinic study,^14^ Washington University Knight Alzheimer Disease Research Centre (ADRC, *n* = 88) study^15^ and European Medical Information Framework for Alzheimer’s Disease Multimodal Biomarker Discovery study (EMIF-AD MBD, *n* = 161).^16^ The study received ethical approval from local medical ethics committees at all participating centres, and all participants provided written informed consent prior to inclusion. For the present study, we included participants without cognitive impairment [NC or subjective cognitive decline (NC)] who had baseline CSF samples and available data of CSF Aβ42 and CSF p-tau levels.

### Neuropsychological assessment

A neuropsychological assessment was administered to all participants, including the Mini-Mental State Examination (MMSE) and neuropsychological tests assessing several cognitive domains. Neuropsychological tests varied across centres. The most commonly used memory tests were the Rey Auditory Verbal Learning Test (administered in the BB-ACL and EMIF-AD MBD studies) and the Free and Cued Selective Reminding Test (used in the Washington University Knight ADRC study). Attention was assessed using the Trail Making Test A, executive functioning with the Trail Making Test B, and language with the Animal Fluency test. Further details on the neuropsychological assessments and *Z*-score calculations are provided elsewhere.^14,16,17^ NC was defined as performance on neuropsychological test within 1.5 standard deviation (SD) of the age-, gender-, and education-adjusted mean.

### CSF protein analysis

CSF collection was performed using lumbar puncture, followed by the CSF centrifugation and storage at −80°C in polypropylene tubes. Untargeted proteomic and peptidomic analyses using tandem mass tag spectrometry were performed at the Neurochemistry Lab of the University of Gothenburg, as previously described.^18-20^ A total of 3102 proteins were quantified. For the final analysis, only proteins with data available in at least one-third of participants within each diagnostic group were retained. In cases where proteins had identical values due to fragment non-specificity, one protein was randomly selected for inclusion.^18^ All analyses were performed according to the manufacturer’s instructions and using two batches (batch one including 203 individuals, batch two including 88 individuals) of reagents by board-certified laboratory technicians who were blinded to clinical information.

Targeted CSF biomarker analyses, including Aβ40, Aβ42, phosphorylated tau (p-tau), total tau (t-tau), neurofilament light (NfL), and neurogranin (Ng), were conducted using validated immunoassays (Supplementary Table 1). In the BB-ACL and EMIF-AD MBD cohorts, levels of Aβ40, Aβ42, NfL, and Ng were centrally analyzed by the Neurochemistry Laboratory at the University of Gothenburg, Sweden. In addition, Aβ42, t-tau and p-tau levels were measured locally. For the Washington University Knight ADRC cohort, all biomarkers, including Aβ40, Aβ42, t-tau, p-tau, NfL and Ng, were measured locally.

### Genetic analysis

Protocols for *APOE* genotyping are described elsewhere.^16,21,22^ Participants were classified as *APOE*-ɛ4 carrier or non-carrier.

In a subset of the cognitively unimpaired participants of the EMIF-AD MBD study (*n* = 145), genotype data (Global Screening Array GSA Illumina, Inc.) was available, which allowed the calculation of polygenic risk score for AD (AD PGRS) using PRSice (v2.3).^23^ AD PGRS were calculated based on a genome-wide association study (GWAS) on AD,^24^ as previously reported.^18^ Analyses with and without *APOE* region were performed.

### Image analysis

MRI data acquisition and preprocessing followed site-specific protocols, and a thorough quality check has been performed. Images were analyzed using Freesurfer (version 5.3.0. for EMIF-AD MB and BB-ACL studies and version 5.0. for the Washington University Knight ADRC study, https://surfer.nmr.mgh.harvard.edu).^25^ Subcortical volumes (including hippocampal volume) were normalized by total intracranial volume.

### Participant classification

Amyloid (A) and tau (T) status were determined using locally measured CSF Aβ42 and p-tau levels, respectively. Biomarker abnormality was defined based on cohort-specific thresholds. Given the differences in assay procedures across centres, Aβ42 thresholds were recalculated independently for each cohort using an unbiased Gaussian mixture modelling approach (Supplementary Table 2). We classified participants with NC as A−T− (controls, *n* = 141), A−T+ (NC-SNAP, *n* = 30), A+T− (*n* = 65), or A+T+ (*n* = 55).

### Pathway enrichment analysis

To explore biological pathways associated with the significant up- or down-regulated proteins, GO enrichment analysis were performed using PANTHER (Protein ANalysis THrough Evolutionary Relationships, version 15.0, Los Angeles, CA, USA).^26^ Statistical significance was determined using Fisher’s exact test with false discovery rate correction according to the Benjamini-Hochberg procedure,^27^ retaining only pathways with an FDR-adjusted *P*-value below 0.05.^28,29^ To simplify interpretation and minimize overlap, related GO terms were consolidated into broader functional categories. Molecular functions and cellular components associated with the significantly up- or downregulated proteins were also explored using GO enrichment analysis and are presented in Supplementary Table. Enriched pathways and categories were independently confirmed using complementary tools, including STRING version 11.0^30^ and ClueGO,^31^ a Cytoscape plug-in.

### Brain cell expression analysis

Brain cell-specific protein expression analyses were conducted with the online database Brain RNA-Seq (http://www.brainrnaseq.org/).^32^ Proteins were labelled as being predominantly produced by a cell type when levels were higher than 40% of the total produced across cell types.

### Gene set expression enrichment in adult human brain regions

We explored whether proteins differing between groups were enriched for specific brain regions using ABAEnrichment analysis.^33^ As brain barriers may impact the CSF composition, we also studied proteins associated with brain barriers functioning. We annotated proteins as indicative of blood-brain barrier (BBB) permeability from references^34-36^ or as highly expressed by the choroid plexus (ChP) of the lateral ventricles according to the Allen Brain Atlas^37^ through Harmonizome.^38^

### Weighted gene co-expression network analysis

Weighted Gene Co-expression Network Analysis (WGCNA) was performed to identify co-expressed protein modules and examine their associations with participant groups and AD biomarkers using the R package WGCNA, as previously described.^39^ Proteins with at least one-third of observations across each participant group were included, resulting in 1377 proteins. The co-expression network was constructed with a soft-thresholding power β of 7, selected based on scale-free topology fit and mean connectivity. Pairwise biweight mid-correlation (bicor) was calculated for all protein pairs to generate a signed adjacency matrix. Hierarchical clustering and the dynamic tree cut method were applied to identify protein modules. Module eigengenes, representing the most representative protein abundance values for each module, were computed. GO annotation of the proteins was conducted using the org.Hs.eg.db and GO.db R packages. Protein identifiers were mapped to corresponding Entrez Gene IDs, and associated GO terms were retrieved, allowing us to assess the distribution of GO terms across modules to define the primary function of each module. The relationship between WGCNA modules and AD biomarkers (local CSF Aβ42 and p-tau *Z*-scores) was explored by computing biweight midcorrelation (bicor) between each module eigengene and clinical variables. Non-parametric tests were applied for comparisons between diagnostic groups, as the data was non-normal (assessed via Shapiro-Wilk and Fligner-Killeen tests). The Kruskal-Wallis test was used to assess module expression differences between groups, followed by the Wilcoxon test for pairwise post-hoc comparisons.

### Statistical analysis

To characterize A−T+ individuals, we compared clinical, CSF and imaging measures between biomarker groups using analyses of covariance (ANCOVA) corrected for age and sex for continuous variables and Chi-square for categorical variables. For the targeted CSF and imaging markers, we transformed the values to *Z*-scores as different methodologies were used in different centres. CSF protein levels from untargeted analyses were normalized according to the mean and standard deviation of the control group (NC A−T−) and compared between AT groups using ANCOVA corrected for age and sex. AD PGRS were compared between groups using linear models. Post hoc, these ANCOVA analyses were also performed adjusted for *APOE*-ɛ4 carriership. Statistical analyses were performed using R 3.6.2 and IBM SPSS Statistics version 26. Two-sided statistical significance was used and set at *P* < 0.05.

## Results

### Sample characteristics

Sample characteristics are presented in Table 1. NC A−T+ were older than controls but similar to NC A+T− and NC A+T+. NC A−T+ were less often *APOE*-ɛ4 carriers compared with NC A+T+, similar to controls and NC A+T−. NC A−T+ had lower memory scores compared with controls and NC A+T+. Hippocampal volumes were larger and CSF Aβ40 and Aβ42 levels were higher in NC A−T+ compared with all other groups. The Aβ42/40 ratio was lower in NC A−T+ compared with controls, and higher compared with NC A+T− and NC A+T+. CSF NfL and Ng levels were similar in NC A−T+ and NC A+T+, while p-tau and t-tau levels were lower in NC A−T+ compared with NC A+T+. Ng, p-tau and t-tau levels were higher in NC A−T+ compared with NC A+T− and controls. NfL levels were higher in NC A−T+ compared with NC A+T−.

**Table 1:** Sample characteristics

	Controls *n* = 141	NC-SNAP *n* = 30	NC A+T− *n* = 65	NC A+T + *n* = 55	p NC-SNAP versus controls	p NC-SNAP versus NC A+T−	p NC-SNAP vs NC A +T+
Age, years	64.7 (8.7)	68.2 (8.3)	65.1 (8.6)	70.8 (7.4)	**0**.**047**	0.102	0.138
Female (%)	80 (57)	18 (60)	33 (51)	29 (53)	0.743	0.402	0.519
Education, years	13.1 (3.6)	13.3 (3.8)	13.6 (3.4)	13.4 (4.0)	0.728	0.707	0.973
*APOE*-ɛ4 carriers (%)	26 (23)	9 (34)	32 (52)	30 (57)	0.277	0.112	**0**.**049**
*APOE* genotype (%)	ɛ2/ɛ3 = 22 (20) ɛ2/ɛ4 = 1 (1) ɛ3/ɛ3 = 64 (57) ɛ3/ɛ4 = 24 (21) ɛ4/ɛ4 = 1 (1)	ɛ2/ɛ3 = 2 (7) ɛ2/ɛ4 = 0 (0) ɛ3/ɛ3 = 16 (59) ɛ3/ɛ4 = 8 (30) ɛ4/ɛ4 = 1 (4)	ɛ2/ɛ3 = 4 (6) ɛ2/ɛ4 = 1 (2) ɛ3/ɛ3 = 26 (42) ɛ3/ɛ4 = 25 (40) ɛ4/ɛ4 = 6 (10)	ɛ2/ɛ3 = 4 (7) ɛ2/ɛ4 = 1 (2) ɛ3/ɛ3 = 19 (36) ɛ3/ɛ4 = 18 (34) ɛ4/ɛ4 = 11 (21)	0.409	0.543	0.176^3^
MMSE	28.8 (1.2)	28.5 (1.5)	28.7 (1.2)	28.4 (1.9)	0.225	0.523	0.705
Memory, *Z* score	0.2 (0.9)	−0.1 (1.0)	0.1 (0.9)	0.4 (0.8)	**0**.**015**	0.074	**0**.**016**
Attention, *Z* score	0.2 (1.0)	0.0 (1.2)	0.3 (1.0)	0.3 (0.7)	0.528	0.492	0.223
Executive Functioning, *Z* score	0.3 (1.0)	0.3 (0.8)	0.2 (1.0)	0.2 (0.8)	0.638	0.639	0.674
Language, *Z* score	0.0 (1.0)	−0.3 (0.8)	−0.0 (1.0)	0.0 (0.8)	0.105	0.110	0.092
CSF Aβ40, *Z* score	0.3 (1.2)	0.8 (1.2)	−0.9 (1.0)	−0.1 (1.6)	0.093	**<0**.**001**	**0**.**011**
CSF Aβ42, *Z* score	0.5 (1.3)	1.1 (1.0)	−1.1 (1.0)	−0.5 (1.0)	**0**.**036**	**<0**.**001**	**<0**.**001**
CSF Aβ42/40 ratio. *Z* score	0.9 (1.0)	0.4 (0.9)	−0.7 (1.3)	−1.5 (0.7)	**0**.**03**	**<0**.**001**	**<0**.**001**
CSF NfL, *Z* score	0.0 (1.5)	0.4 (1.2)	−0.4 (1.3)	0.2 (1.1)	0.154	**0**.**007**	0.512
CSF Ng, *Z* score	−0.3 (0.7)	1.0 (3.0)	−0.5 (0.5)	0.6 (1.4)	**<0**.**001**	**<0**.**001**	0.568
CSF P-tau, *Z*-score	−0.4 (0.5)	0.8 (0.8)	−0.5 (0.5)	1.3 (1.2)	**<0**.**001**	**<0**.**001**	**0**.**026**
CSF T-tau, *Z*-score	−0.3 (0.5)	0.5 (0.8)	−0.5 (0.6)	1.2 (1.4)	**<0**.**001**	**<0**.**001**	**0**.**015**
Hippocampal volume, *Z*-score	0.1 (1.3)	0.9 (1.6)	−0.3 (1.5)	−0.4 (1.3)	**0**.**012**	**0**.**005**	**<0**.**001**

Values represent mean (standard deviation) or number (percentages). Significant *P*-values (<0.05) are bold. The sample size was smaller for some variables: 37 values are missing for APOE genotype. 14 values are missing for memory, 43 values for attention and language, and 45 for executive functioning. 39 values are missing for Aβ40, Aβ42, Aβ42/40 ratio and NfL. 42 values are missing for Ng and 41 for hippocampal volume. T-tau and p-tau values were measures locally and so are presented as *Z*-scores with controls within each data set as a reference. Results for comparison of APOE genotypes were: 1 = The percentage of ɛ2ɛ3 carrier is higher in controls compared with NC A+T− (*P* = 0.046); The percentage of ɛ3ɛ4 carrier is lower in controls compared with NC A+T− (*P* = 0.008); The percentage of ɛ4ɛ4 carrier is lower in controls compared with NC A+T− (*P* = 0.005). 2 = The percentage of ɛ2ɛ3 carrier is higher in controls compared with NC A+T+ (*P* = 0.046); The percentage of ɛ3ɛ3 carrier is higher in controls compared with NC A+T+ (*P* = 0.011); The percentage of ɛ4ɛ4 carrier is lower in controls compared with NC A+T+ (*P* = <0.001). 3 = The percentage of ɛ3ɛ3 carrier is higher in NC-SNAP compared with NC A+T+ (*P* = 0.046); The percentage of ɛ4ɛ4 carrier is lower in NC-SNAP compared with NC A+T+ (*P* = 0.043).

Abbreviations: A, amyloid; Aβ, amyloid β; AD, Alzheimer’s disease; MMSE, Mini Mental State Examination; NC, normal cognition; NfL, neurofilament light; Ng, neurogranin; *P*, *P*-value; P-tau, phosphorylated tau; SNAP, suspected non-Alzheimer’s pathophysiology; T, tau; T-tau, total tau.

We also examined the differences in AD PGRS between the groups in a subset of individuals from the EMIF-AD MBD study (*n* controls = 65; *n* NC A−T+ = 12; *n* NC A+T− = 44, *n* NC A+T+ = 24). We found significantly higher AD PGRS in NC A+T− and A+T+ individuals compared with controls for the threshold *P*-value of 1e−30, 1e−8 and 5e−8, and, only for A+T+, also for threshold 1e−5 (Supplementary Fig. 1A). NC A−T+ showed significantly higher AD PGRS compared with controls for the threshold *P*-value of 1e−30 (Supplementary Fig. 1A). Differences between the groups were mainly driven by *APOE* (Supplementary Fig. 1B). No significant AD PGRS differences were found between NC A−T+, NC A+T− and NC A+T+. Age and sex correction did not change the results (Supplementary Table 3A and B).

### CSF proteomic profiles of NC A−T+, A+T− and A+T+ compared with controls

**Figure 1:**
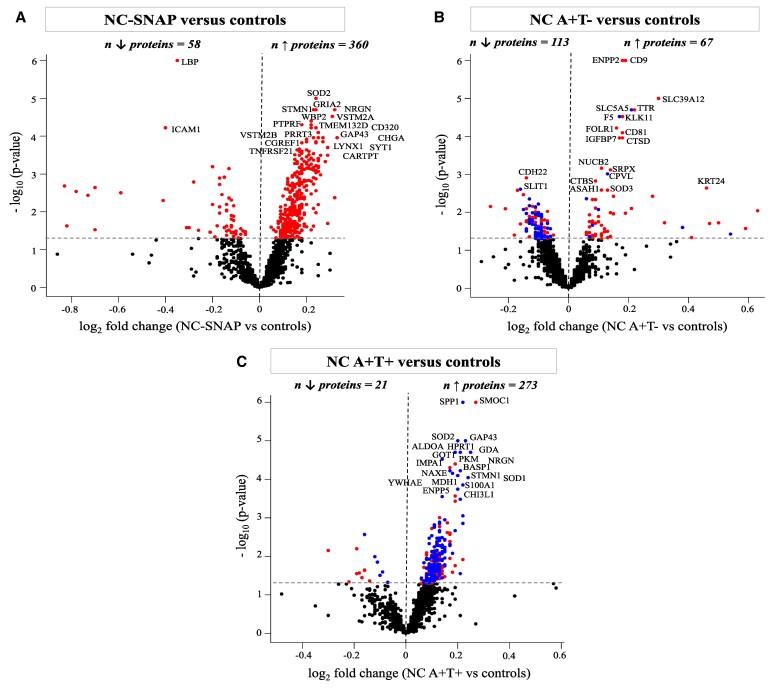
**Differential levels of CSF proteins across groups.** (**A–C**) Volcano plots showing the CSF proteins with significantly different levels in NC A−T+, NC A+T−, or NC A+T+ versus controls. CSF protein levels were compared between groups using ANCOVA corrected for age and sex. Each dot represents a protein. The log_2_ fold change are plotted versus the −log_10_  *P*-values. For graphs (**B**) and (**C**), significant proteins overlapping with the different proteins in the comparison NC A−T+ versus controls are in blue. The name of the top 20 significantly different CSF proteins are annotated. The total number of proteins that are decreased (left) or increased (right) is indicated. Horizontal dotted line indicates the significance threshold. NC, normal cognition; SNAP, Suspected non-Alzheimer’s disease pathophysiology (A−T+); A+, abnormal levels of CSF Aβ42; T+, abnormal levels of CSF phosphorylated-tau.

**Figure 2:**
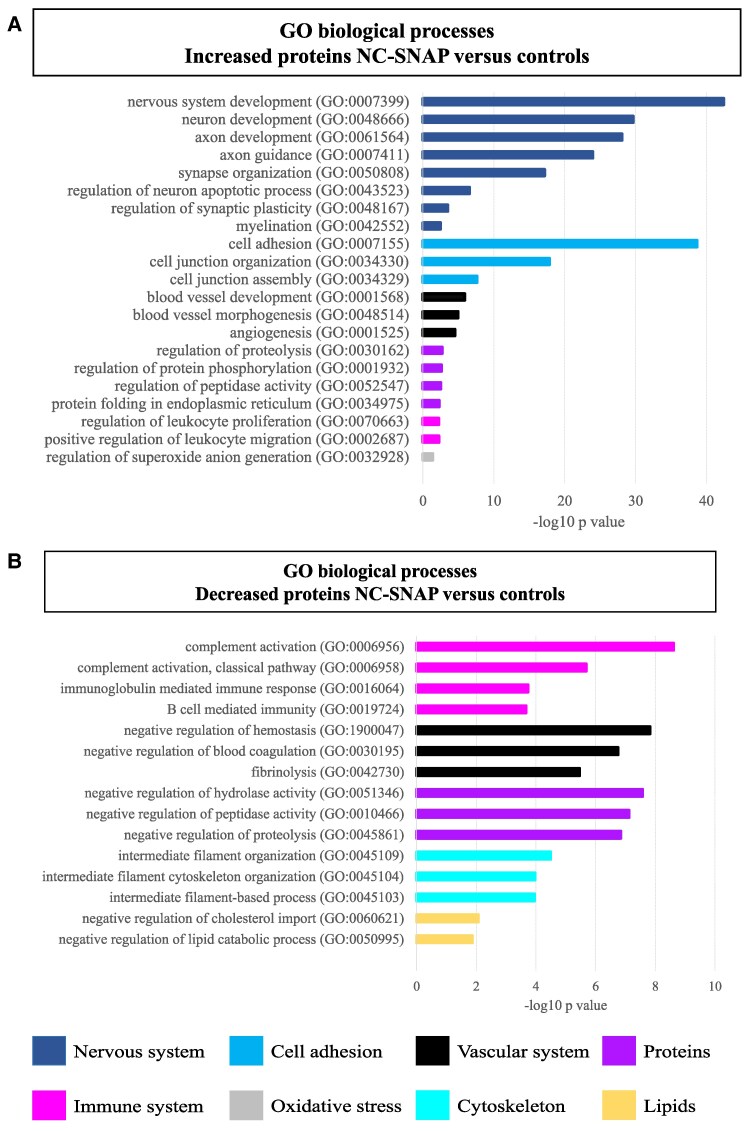
**CSF proteomics in NC-SNAP (A−T+) compared with controls.** (**A**) Selected biological processes GO terms for the increased proteins in the comparison NC A−T+ versus controls. (**B**) Selected biological processes GO terms for the decreased proteins in the comparison NC A−T+ versus controls. Pathway enrichment analyses were performed using Fisher’s exact test with false discovery rate (using the Benjamini-Hochberg procedure). GO, Gene Ontology; NC, normal cognition; SNAP, Suspected non-Alzheimer’s disease pathophysiology (A−T+); A+, abnormal levels of CSF Aβ42; T+, abnormal levels of CSF phosphorylated-tau; NC, normal cognition; SNAP, Suspected non-Alzheimer’s disease pathophysiology (A−T+); A+, abnormal levels of CSF Aβ42; T+, abnormal levels of CSF phosphorylated-tau.

In NC A−T+, 360 proteins were increased and 58 decreased compared with controls (Fig. 1A, Supplementary Table 4A). The increased proteins in NC A−T+ were mainly enriched for biological processes linked to the nervous system, cell adhesion, angiogenesis, protein modification and degradation, and immune system (Fig. 2A, Supplementary Table 4B). The cell mapping analysis indicated that increased proteins were predominantly expressed in the neurons (129 proteins, 36%). The decreased proteins in NC A−T+ were enriched for biological processes related to the immune system, hemostasis, proteolysis, cytoskeleton and lipids (Fig. 2B, Supplementary Table 4B). Of the decreased proteins, 33% (19 proteins) were enriched for expression in the ChP (ABAenrichment *P* = 0.065) and 43% (25 proteins) were associated with BBB functioning.

In NC A+T−, 67 proteins were increased and 113 decreased compared with controls (Fig. 1B, Supplementary Table 4A and E), while in NC A+T+, 273 proteins were increased and 21 decreased compared with controls (Fig. 1C, Supplementary Table 4A and F), as detailed in Supplementary Results.

**Figure 3:**
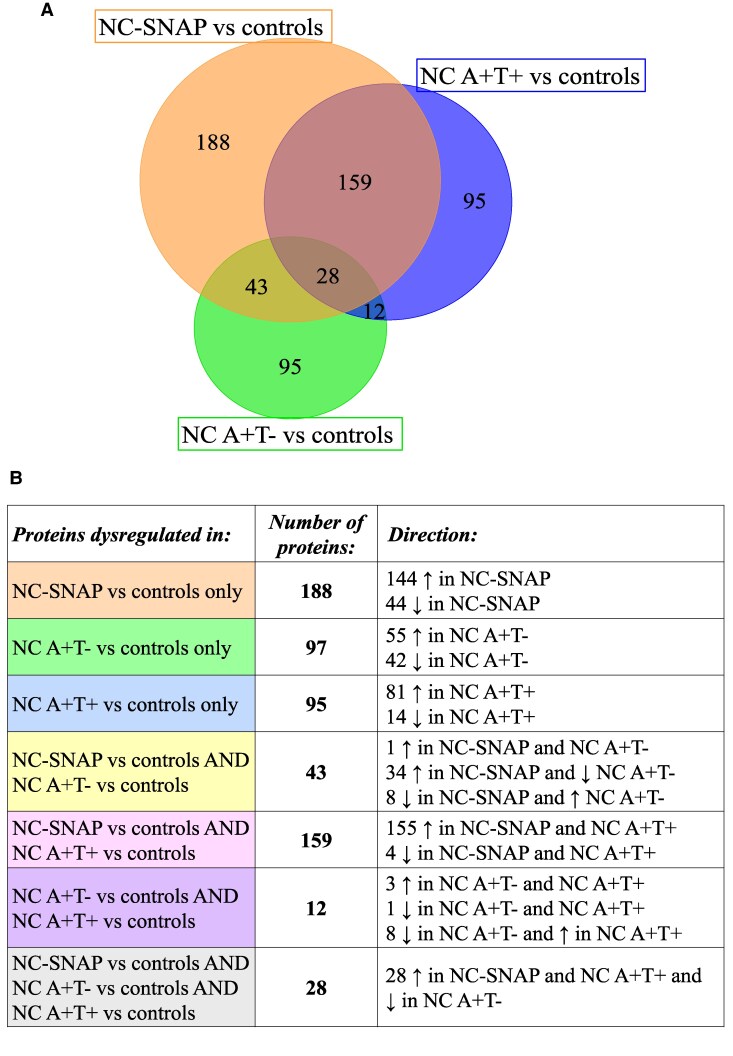
**Overlap of significantly dysregulated CSF proteins in NC A/T groups versus controls.** (**A**) Proportional Venn diagram showing the overlap in proteins with significantly different levels between NC A−T+ and controls as well as between NC A+T− and controls and between NC A+T+ and controls. (**B**) Table depicting the number and direction of the proteins with significantly different levels from the comparisons shown in the Venn diagram.

We next compared the proteins with significantly different levels in the different biomarker group comparisons with controls (Fig. 3A and B). Out of the 418 proteins with significantly different levels in NC A−T+ versus controls, ∼40% (159 proteins) were also significantly different in NC A+T+ compared with controls, mainly in the same direction and upregulated (Fig. 3A and B). These proteins were associated with biological pathways linked to the nervous system, oxidative stress and energy metabolism and were predominantly expressed in the neurons (60 proteins, 39%). Approximatively half (188 proteins) of the proteins with significantly different levels in NC A−T+ versus controls were uniquely different in NC A−T+ (Fig. 3A and B). The increased proteins were mainly associated with cell adhesion, nervous system, proteins, vascular system and immune system, while the decreased proteins were mainly associated with immune system, proteolysis, cytolysis, cytoskeleton and, hemostasis and fibrinolysis, and were associated with BBB functioning (20 proteins, 45%). Of the proteins with significantly different levels in NC A+T− versus controls, 83 proteins (47%) were also significantly different in NC A−T+ and/or NC A+T+ versus controls, albeit mainly in the opposite direction in NC A+T− compared with NC A−T+ and NC A+T+ (Fig. 3A and B).

### Comparisons between CSF proteomic profiles of NC A−T+, A+T− and A+T+

To study more thoroughly the proteomic differences between SNAP and AD, we next directly compared CSF proteins in NC A−T+ with NC A+T− and NC A+T+.

**Figure 4:**
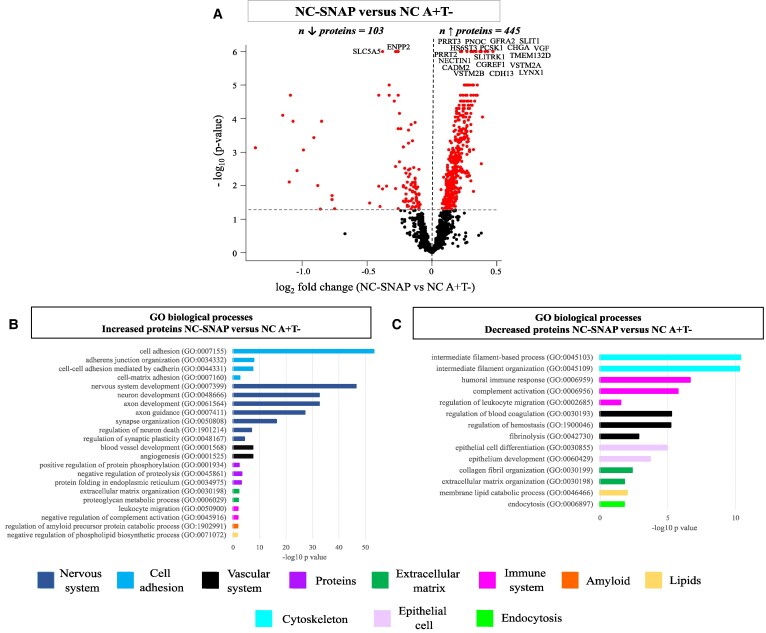
**CSF proteomics in NC-SNAP (A−T+) compared with NC A+T−.** (**A**) Volcano plots showing the CSF proteins that were significantly different in NC A−T+ versus NC A+T−. CSF protein levels were compared between groups using ANCOVA corrected for age and sex. Each dot represents a protein. The log2 fold change are plotted versus the −log10 *P*-values. The name of the top 20 significant CSF proteins are annotated. The total number of proteins that are decreased (left) or increased (right) is indicated. Horizontal dotted line indicates the significance threshold. (**B**) Selected biological processes GO terms for the increased proteins in the comparison NC A−T+ versus NC A+T−. (**C**) Selected biological processes GO terms for the decreased proteins in the comparison NC A−T+ versus NC A+T−. Pathway enrichment analyses were performed using Fisher’s exact test with false discovery rate (using the Benjamini-Hochberg procedure). GO, Gene Ontology; NC, normal cognition; SNAP, Suspected non-Alzheimer’s disease pathophysiology (A−T+); A+, abnormal levels of CSF Aβ42; T+, abnormal levels of CSF phosphorylated-tau.

Comparing NC A−T+ to NC A+T−, 445 proteins were increased and 103 decreased (Fig. 4A, Supplementary Table 4A). The increased proteins were mainly enriched for biological processes linked to cell adhesion, nervous system, angiogenesis, proteins and extracellular matrix (ECM) (Fig. 4B, Supplementary Table 4C). Increased proteins were predominantly expressed by the neurons (161 proteins, 36%). The decreased proteins were mainly enriched for biological processes related to the cytoskeleton, immune system, proteolysis, hemostasis and fibrinolysis, and epithelial cell (Fig. 4C, Supplementary Table 4C). Forty-seven percent (48 proteins) of the decreased proteins were enriched for expression in the ChP (ABAenrichment *P* = <0.001) and twenty-three percent were associated with BBB functioning (24 proteins).

**Figure 5:**
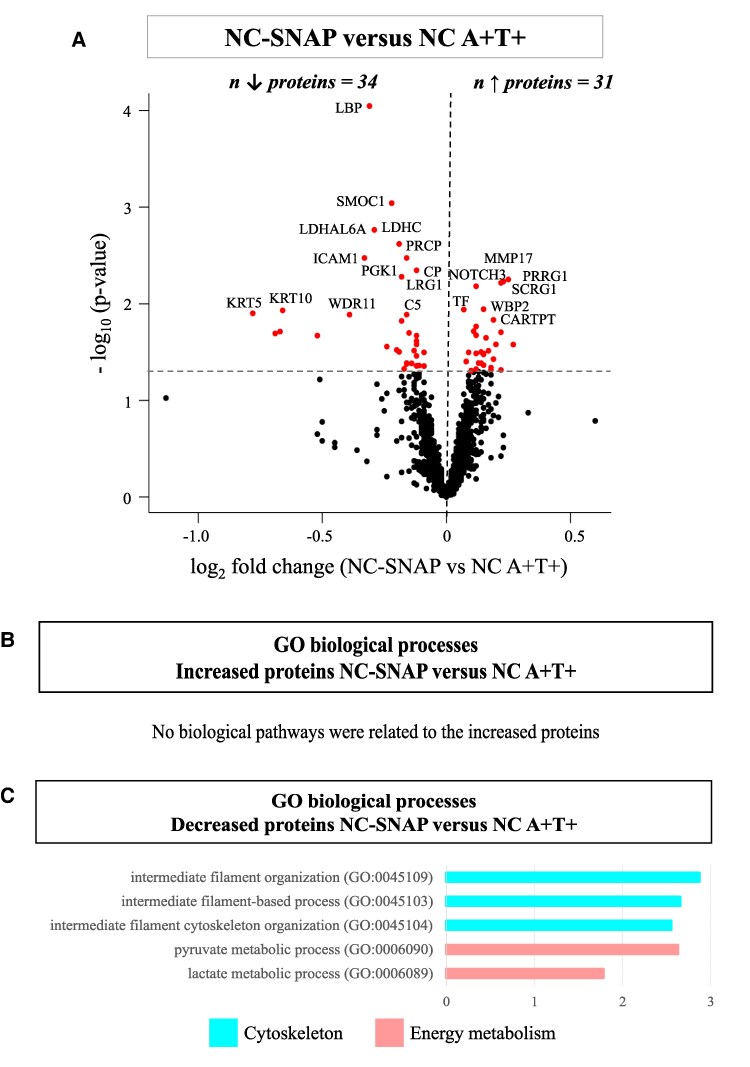
**CSF proteomics in NC-SNAP (A−T+) compared with NC A+T+.** (**A**) Volcano plots showing the CSF proteins that were significantly different in NC A−T+ versus NC A+T+. CSF protein levels were compared between groups using ANCOVA corrected for age and sex. Each dot represents a protein. The log2 fold change are plotted versus the −log10 *P*-values. The name of the top 20 significant CSF proteins are annotated. The total number of proteins that are decreased (left) or increased (right) is indicated. Horizontal dotted line indicates the significance threshold. (**B**) Selected biological processes GO terms for the increased proteins in the comparison NC A−T+ versus NC A+T+. (**C**) Selected biological processes GO terms for the decreased proteins in the comparison NC A−T+ versus NC A+T+. Pathway enrichment analyses were performed using Fisher’s exact test with false discovery rate (using the Benjamini-Hochberg procedure). GO, Gene Ontology; NC, normal cognition; SNAP, Suspected non-Alzheimer’s disease pathophysiology (A−T+); A+, abnormal levels of CSF Aβ42; T+, abnormal levels of CSF phosphorylated-tau.

Comparing NC A−T+ to NC A+T+, 31 proteins were increased and 34 decreased (Fig. 5A, Supplementary Table 4A). The increased proteins were not associated with specific biological processes (Fig. 5B), but were predominantly expressed by the neurons (29%, 9 proteins). The decreased proteins were enriched for biological processes linked to cytoskeleton and energy metabolism (Fig. 5C, Supplementary Table 4D). Forty-four percent (15 proteins) of the decreased proteins were enriched for expression in the ChP (ABAenrichment *P* = 0.314).

### Protein co-expression network

Protein co-expression network analysis using WGCNA identified 9 modules, ranging in size from 538 (M1) to 23 (M9) proteins. GO analysis of the protein module members revealed distinct functional annotations for all 9 modules (Supplementary Fig. 2A). Four modules were significantly correlated with CSF Aβ42 and/or p-tau levels (Supplementary Fig. 2A). Module M1, associated with nervous system development, showed a positive correlation with both CSF Aβ42 and p-tau levels. Module M7, related to immune response, was negatively correlated with both CSF Aβ42 and p-tau levels. Module M6, related to immunoglobulins, was negatively correlated with CSF p-tau only, while Module M5, related to complement, was negatively correlated with CSF Aβ42 only.

When examining differences in module eigengene values between groups, four modules showed significant differences (Supplementary Fig. 2B). Module M1, associated with nervous system development, was upregulated in NC A−T+ and NC A+T+ compared with controls and NC A+T−. Module M2, related to synapse function, was upregulated in NC A+T+ versus controls and A+T−. Module M5, related to complement, was downregulated in NC A−T+ compared with A+T− and showed a trend towards significance compared with A+T+ (*P*-value = 0.07). Finally, Module M7, related to immune response, was increased in NC A+T− compared with all other groups.

### CSF proteomics to identify unique processes in NC A−T+

**Figure 6:**
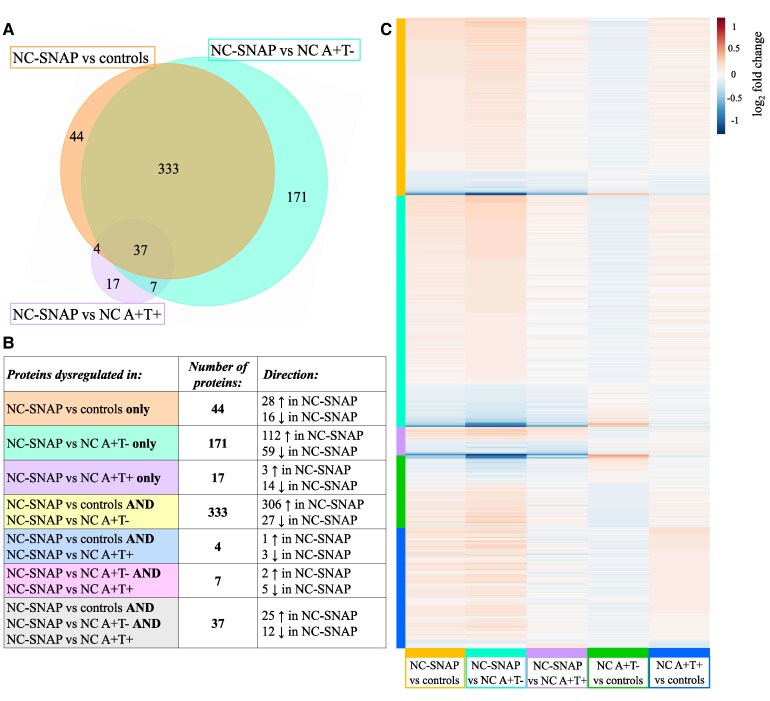
**CSF proteomic profiles of the different comparisons and proteins uniquely associated with NC-SNAP (A−T+).** (**A**) Proportional Venn diagram showing the overlap in proteins with significantly different levels between NC A−T+ and controls, between NC A−T+ and NC A+T− and between NC A−T+ and NC A+T+. The total number of proteins with significantly different levels in each of the groups is annotated. (**B**) Table depicting the number and direction of the different proteins as showed in the Venn diagram. (**C**) Heatmap representing the log2 fold-change values of the proteins with significantly different levels in each comparison. Statistical analysis was performed using ANCOVA corrected for age and gender. Significant differences were determined based on *P*-values < 0.05. NC, normal cognition; SNAP, Suspected non-Alzheimer’s disease pathophysiology (A−T+); A+, abnormal levels of CSF Aβ42; T+, abnormal levels of CSF phosphorylated-tau.

To identify the uniquely different proteins and pathways in NC A−T+, we selected the significantly different proteins overlapping in NC A−T+ versus controls, NC A−T+ versus NC A+T− and NC A−T+ versus NC A+T+ comparisons (Fig. 6A–C). This resulted in a total of 37 proteins uniquely associated with NC A−T+, in which 25 were increased and 12 were decreased (Fig. 6A and B). All together, these proteins were linked with the cytoskeleton, iron ion transport and homeostasis and acute inflammatory response. The increased proteins were not linked with clear biological processes but were predominantly produced by the neurons (28%, 7 proteins). In the literature, a significant proportion of the increased proteins have shown to be related to neuroplasticity (ACE, BDNF, CARTP, CCK, LYNX1, NOTCH3 and VGF), proteolysis (MANEAL, MMP17, UBQLN2) and iron uptake and transport (TF and TFRC). The decreased proteins were associated with biological pathways associated with the cytoskeleton and epithelial cells (Supplementary Table 4G). Approximatively half of the decreased proteins were keratins (KRT1, KRT2, KRT5, KRT9 and KRT10). In the literature, other decreased proteins have been shown to be associated with the immune system (C5, ICAM1 and LBP), iron homeostasis and transport (CP and HPX) and proteolysis (CPVL and PRLP). Fifty-eight percent (7 proteins) of the decreased proteins were enriched for expression in the ChP (ABAenrichment *P* = 0.398).

### Post hoc analysis

To better understand the influence of *APOE*-ɛ4 on our CSF findings, we also performed analyses with *APOE*-ɛ4 correction, which did not appreciably change the results (Supplementary Table 5).

Moreover, we also corrected our analyses for batch effects, which resulted in similar findings. We also corrected our analysis for centre-specific effects, which resulted in similar results. As EMIF-AD MBD and Maastricht BB-ACL data were acquired in batch 1, while Washington University Knight ADRC data were acquired in batch 2, we conducted separate analyses for the two batch cohorts to validate our findings within each cohort independently. The results were consistent with our original findings, supporting the robustness of our conclusions.

## Discussion

This study investigated the pathophysiology of NC-SNAP (A−T+) in relation to preclinical Alzheimer’s disease (NC A+T− and NC A+T+) and controls using CSF proteomics. We found that NC A−T+ showed mainly increased protein levels compared with controls, which were associated with neuroplasticity, angiogenesis, protein modification and degradation, and immune system. The proteomic profile of NC A−T+ was relatively similar to that of NC A+T+, but distinct from that of NC A+T−, which showed mainly decreased proteins related to neuroplasticity. Together, these results indicate that the CSF proteomic profile of both NC A−T+ and NC A+T+ are mainly driven by tau rather than amyloid.

Compared with controls, NC A−T+ showed mostly increased levels of proteins, which were associated with neuroplasticity, angiogenesis, protein modification and degradation, and leukocytes. These protein changes are likely driven by tau pathophysiology. Neuronal plasticity might reflect a compensatory response due to tau-induced synaptic dysfunction.^23,40^ Angiogenesis could also be part of this neuronal plasticity response.^23,41^ The increased levels of proteins associated with protein phosphorylation and proteolysis may also be linked with tau hyperphosphorylation^42^ and tau proteolysis.^43^ Finally, increased tau levels can lead to neuroinflammation, including activation of microglia and astrocytes, which could enhance the transport of leukocytes across the BBB.^42,44^ A recent study suggests an alternative explanation for the increased levels of proteins in NC-SNAP, indicating that some A−T+ individuals may present non-disease-related increases in overall CSF protein levels.^45^ This inherent variability could contribute to the increased protein levels observed in NC-SNAP.

The decreased proteins in NC A−T+ compared with controls, while not predominant, were associated with pathways linked to immune system, hemostasis, proteolysis, cytoskeleton (intermediate filament) and lipids. These proteins changes may be associated with ChP and BBB functioning,^23,46^ which both have been shown to be compromised with aging and AD.^47^ Indeed, the ChP is a highly vascularized structure composed of specialized epithelial cells that contain intermediate filaments.^48^ It plays an important role in transport of lipids across the epithelium into the CSF, and in protein clearance, such as Aβ, from the CSF. It has also important immune functions, as it is a gateway for immune cell entry into the brain and contains and activate complement components.^49-54^ Moreover, pathways associated with complement, immunoglobulins and B cells, blood coagulation and lipids have previously been associated with disturbed BBB functioning.^23^ These pathway associations with ChP and BBB functioning were shown previously in SNAP and Alzheimer’s disease,^18,23,46^ although proteins were increased in Alzheimer’s disease.

Comparing NC A−T+ to NC A+T+, minimal proteomic differences were found, suggesting that the observed proteomic profiles are more related to tau than to amyloid. Both groups exhibited predominantly increased levels of plasticity-related proteins compared with controls, with considerable overlap. However, amyloid markers in NC-SNAP were clearly in the normal range, and according to established definitions, the absence of amyloid means no AD. Additionally, the frequency of *APOE*-ɛ4 carriers was lower in NC A−T+ compared with NC A+T+, which aligns with previous studies.^2,8^ The latter could explain the absence of Aβ aggregation in NC-SNAP^55^ and indicates that NC-SNAP is biologically different from typical AD. NC-SNAP may represent primary age-associated tauopathy, which is characterized by pathology that is similar to neurofibrillary tangles in preclinical Alzheimer’s disease.^56^ Persons with NC-SNAP may be resistant to amyloid, although amyloid resistance is often associated with specific genetic variants.^57^ Nonetheless, apart from *APOE*, our study did not support this notion. Future genetic studies are needed to validate that hypothesis.

NC A−T+ showed a different proteomic profile from NC A+T−. NC A+T− showed mainly decreased levels of plasticity proteins. Overlapping proteins (40%) were mostly increased in NC A−T+ and decreased in NC A+T− and were mainly associated with neuroplasticity and influenced by tau.^23^ ChP dysfunction was associated with both NC A−T+ and NC A+T−, although related to distinct proteins and changes in opposite directions. The observed decreased levels in plasticity-related proteins and increased levels of proteins linked with ChP dysfunction in A+T− have been shown previously.^23^ Therefore, it seems that both amyloid and tau abnormality drive changes in neuroplasticity and ChP functioning but in a different way. Unlike in NC A−T+, decreased proteins in NC A+T− were not associated with BBB-related protein changes.

We identified 37 proteins uniquely dysregulated in SNAP (A−T+). Some are plasticity-related proteins and higher levels of those proteins are known to be linked with less Aβ accumulation or better cognitive outcomes.^58-64^ Some protein changes were associated with iron, and previous papers reported an association with iron accumulation and tau pathology in AD.^65,66^ Moreover, several proteins were keratins. This could be linked with changes in ChP functioning, as ChP epithelial cells contain keratins.^48^ Furthermore, we observed higher levels of Aβ40 and Aβ42 in NC A−T+ compared with all other groups. This may indicate disrupted amyloid metabolism, with either increased production or decreased clearance, which could result from changes in ChP functioning.^45,52^ Of note, NC-SNAP A−T+ individuals exhibited larger hippocampal volumes compared with all other AT groups, which suggests that NC-SNAP A−T+ may be different from NC-SNAP A−N+, based on smaller hippocampal volume.

SNAP is a biomarker-concept independent of the clinical status, and therefore can also occur in MCI. In contrast to the increased protein levels in NC-SNAP, in our previous proteomic study, we found mainly decreased protein levels in MCI-SNAP compared with controls.^18^ These proteins were associated with different biological pathways than in NC-SNAP, with mainly some differences ECM-related pathways. However, the decreased proteins partially overlapped (21%) and were related to hemostasis, immune system, lipids and proteolysis, and with a predominant expression in the ChP.^18^ Overall, it seems that the pathophysiological profile of SNAP is dependent on clinical status. Cognitive impairments in SNAP may result from various comorbid non-Alzheimer’s disease pathologies,^67-69^ leading to a distinct underlying pathology than in NC-SNAP. NC-SNAP may reflect a more pure form of tau pathology such as primary age-associated tauopathy (PART) or atypical Alzheimer’s disease.

Our study has several strengths and limitations. This study introduces novel insights into the pathophysiological characterisation of NC-SNAP (A−T+) and provides a better understanding of the differences and similarities between NC-SNAP and preclinical Alzheimer’s disease. The use of large-scale proteomics alongside a substantial sample size enables robust statistical power and a thorough pathophysiological characterisation of the groups. Similar to many prior studies on SNAP, we defined groups based on tau using CSF p-tau181, while the use of alternative p-tau variants, such as p-tau217 and p-tau231, to define SNAP may yield distinct pathophysiological profiles. SNAP can also be defined using tau positron emission tomography imaging, potentially representing a different subset of individuals. Indeed, a previous study showed that A−T+ individuals (stratified by CSF measurement) had similar tau-PET tracer retention in temporal areas as the A−T− group,^10^ suggesting that CSF tau and tau PET do not correlate in A−T+ individuals. Furthermore, there is currently no evidence indicating that tau-PET tracers are able to capture PART, which may be relevant to understanding NC-SNAP. SNAP can also be defined using neurodegeneration markers, such as hippocampal volume or CSF neurofilament, which could result in different SNAP prevalence as well as proteomic profiles. Furthermore, revised criteria for diagnosis, staging and biomarker classification in AD are currently in development,^70^ which may influence the classification and identification of SNAP cases. Given the exploratory nature of these findings, interpretations of the proteomic profiles and related pathways in NC-SNAP should be considered provisional, pending validation in larger, independent cohorts and through a more holistic approach. Further research is also needed to elucidate the causes and consequences of the dysregulated processes associated with NC-SNAP. Longitudinal studies are needed to investigate cognitive outcomes and longitudinal changes in proteomic profiles in NC-SNAP and compared them with those of preclinical Alzheimer’s disease.

Our study initiates the characterisation of the underlying pathophysiology of NC-SNAP (A−T+), showing altered levels of proteins involved in neuronal plasticity, angiogenesis, protein modification and degradation, and immune system. We found a similar CSF proteomic profile in NC-SNAP and NC A+T+, suggesting that tau, rather than amyloid, may be the main driver of the CSF proteomic profiles in NC-SNAP and other AT groups. This may have implications for future proteomic studies and clinical trial design, underscoring the need of considering tau status in future research and trials.

## Supplementary Material

fcaf253_Supplementary_Data

## Data Availability

The EMIF-AD MBD mass spectrometry proteomics data have been deposited to the ProteomeXchange Consortium via the PRIDE partner repository with the dataset identifiers PXD019910 and 10.6019/PXD019910. The codes used for the proteomic analyses are available at: https://github.com/AuroreDelvenne/NC-SNAP_code.
