# Supplementary material for: Cerebrospinal fluid proteomic profiling of cognitively unimpaired individuals with suspected non-Alzheimer's disease pathophysiology
Source: Brain Commun. 2025 Jun 20;7(4):fcaf253. doi: 10.1093/braincomms/fcaf253 (PMC12242384; doi:10.1093/braincomms/fcaf253)
Supplement: fcaf253_Supplementary_Data [file fcaf253_supplementary_data.zip › Supplementary_material (including Supplementary Tables 1 and 2).docx]

**CSF proteomic profiling of cognitively unimpaired individuals with suspected non-Alzheimer's disease pathophysiology**

**Supplement**

Aurore Delvenne, Johan Gobom, Lianne M. Reus, Valerija Dobricic, Mara ten Kate, Suzanne E. Schindler, Inez Ramakers, Betty M. Tijms, Rik Vandenberghe, Jolien Schaeverbeke, Pablo Martinez-Lage, Mikel Tainta, Charlotte E. Teunissen, Julius Popp, Gwendoline Peyratout, Magda Tsolaki, Yvonne Freund-Levi, Simon Lovestone, Johannes Streffer, Frederik Barkhof, Lars Bertram, Kaj Blennow, Henrik Zetterberg, Pieter Jelle Visser and Stephanie J.B. Vos.

**Supplementary Results**

**CSF proteomic profiling of NC A+T- versus controls**

In NC A+T-, 67 proteins were increased and 113 decreased compared to controls (Supplementary Table 4A). The increased proteins in NC A+T- were enriched for biological processes associated with the cytoskeleton (intermediate filament), sphingolipids, cell migration and epithelial cells (Supplementary Table 4E). Fifty-five percent (37 proteins) of the increased proteins were enriched for expression in the ChP (ABAenrichment *P* = <0.001). The decreased proteins in NC A+T- were enriched for biological processes linked to the nervous system, cell adhesion, angiogenesis, epithelial cells, protein processing, amyloid-β and phospholipids (Supplementary Table 4E). Decreased proteins were predominantly expressed by the neurons (38%, 43 proteins).

**CSF proteomic profiling of NC A+T+ versus controls**

In NC A+T+, 273 proteins were increased and 21 decreased compared to controls (Supplementary Table 4A). The increased proteins in NC A+T+ were enriched for biological processes associated with the nervous system, cell adhesion, energy metabolism, amyloid-β, proteolysis and protein phosphorylation, oxidative stress, mitochondrion transport and actin (Supplementary Table 4F). The increased proteins were predominantly expressed by the neurons (105 proteins, 39%). The decreased proteins in NC A+T+ were enriched for biological processes linked to the immune system (immunoglobulins and B cells) and hemostasis (Supplementary Table 4F). Twenty-four percent (5 proteins) of the decreased proteins were associated with BBB permeability.

**Supplementary figures and tables**

**Supplementary Table 1. Summary of targeted markers and methods by cohort.**

**Supplementary Table 2. Center-specific Aβ42 and p-tau cut-off point.**

**Supplementary Table 3. Estimated means (sd) of AD GWAS-based polygenic risk scores in controls, NC-SNAP, NC A+T- and NC A+T+.**

**Supplementary Table 4. Dysregulated proteins for each comparison in the whole list of identified proteins (threshold of 1/3 observations per group) and gene ontology (GO) term enrichment for dysregulated proteins.**

**Supplementary Table 5. Dysregulated proteins for each comparison in the whole list of identified proteins (threshold of 1/3 observations per group), with *APOE*-ε4 correction.**

**Supplementary Figure 1. Polygenic risk score (PGRS) across single nucleotide polymorphism (SNP) thresholds.**

**Supplementary Figure 2. Protein Network Analysis.**

| **Supplementary Table 1. Summary of targeted markers and methods by cohort.** | | | |
| --- | --- | --- | --- |
| **Markers** | **Methods** | | |
|  | **EMIF-AD MBD and BB-ACL** | | **Washington University Knight ADRC** |
| ***Aβ40*** | Central | V-PLEX Plus Aβ Peptide Panel 1 (6E10) Kit, Meso Scale Discovery (MSD, Rockville, MD) | LUMIPULSE G1200, Fujirebio, Malvern, PA |
| ***Aβ42*** | Central | V-PLEX Plus Aβ Peptide Panel 1 (6E10) Kit, Meso Scale Discovery (MSD, Rockville, MD) | LUMIPULSE G1200, Fujirebio, Malvern, PA |
|  | Local | INNOTEST ELISAS (Fujirebio, Ghent, Belgium) or Alzbio3 xMAP Luminex |  |
| ***NfL*** | Central | NF-light® ELISA, UmanDiagnostics, Umeå, Sweden | NF-light® ELISA, UmanDiagnostics |
| ***Ng*** | Central | in-house immunoassay | Single Molecule Counting (SMC™) and Singulex Erenna® platform |
| ***P-tau*** | Local | INNOTEST ELISAS (Fujirebio, Ghent, Belgium) or Alzbio3 xMAP Luminex | LUMIPULSE G1200, Fujirebio, Malvern, PA |
| ***T-tau*** | Local | INNOTEST ELISAS (Fujirebio, Ghent, Belgium) or Alzbio3 xMAP Luminex | LUMIPULSE G1200, Fujirebio, Malvern, PA |
| Abbreviations: Aβ = amyloid; NfL = neurofilament light; Ng = neurogranin; P-tau = phosphorylated tau; T-tau = total tau. | | | |

| **Supplementary Table 2. Center-specific Aβ42 and p-tau cut-off point.** | | |
| --- | --- | --- |
| **Centers** | **cut-off CSF Aβ42 (pg/ml)** | **cut-off CSF p-tau (pg/ml)** |
| Amsterdam [1] | *<813* | *>52* |
| Antwerp [2] | *<669* | *>56.5* |
| DESCRIPA [3] | *<530* | *>52* |
| EDAR [4]* | *<316* | *>35* |
| GAP [5] | *<655* | *>61* |
| Lausanne [6] | *<752* | *>55* |
| Leuven [7] | *<626* | *>80* |
| Maastricht [8] | *<775* | *>52* |
| WashU Knight ADRC [9] | *<588* | *>44.3* |
| *EDAR used Luminex assay and the other centers used ELISA. References are given for each center. Abbreviations: CSF = cerebrospinal fluid, Aβ42 = amyloid beta, p-tau = phosphorylated tau | | |


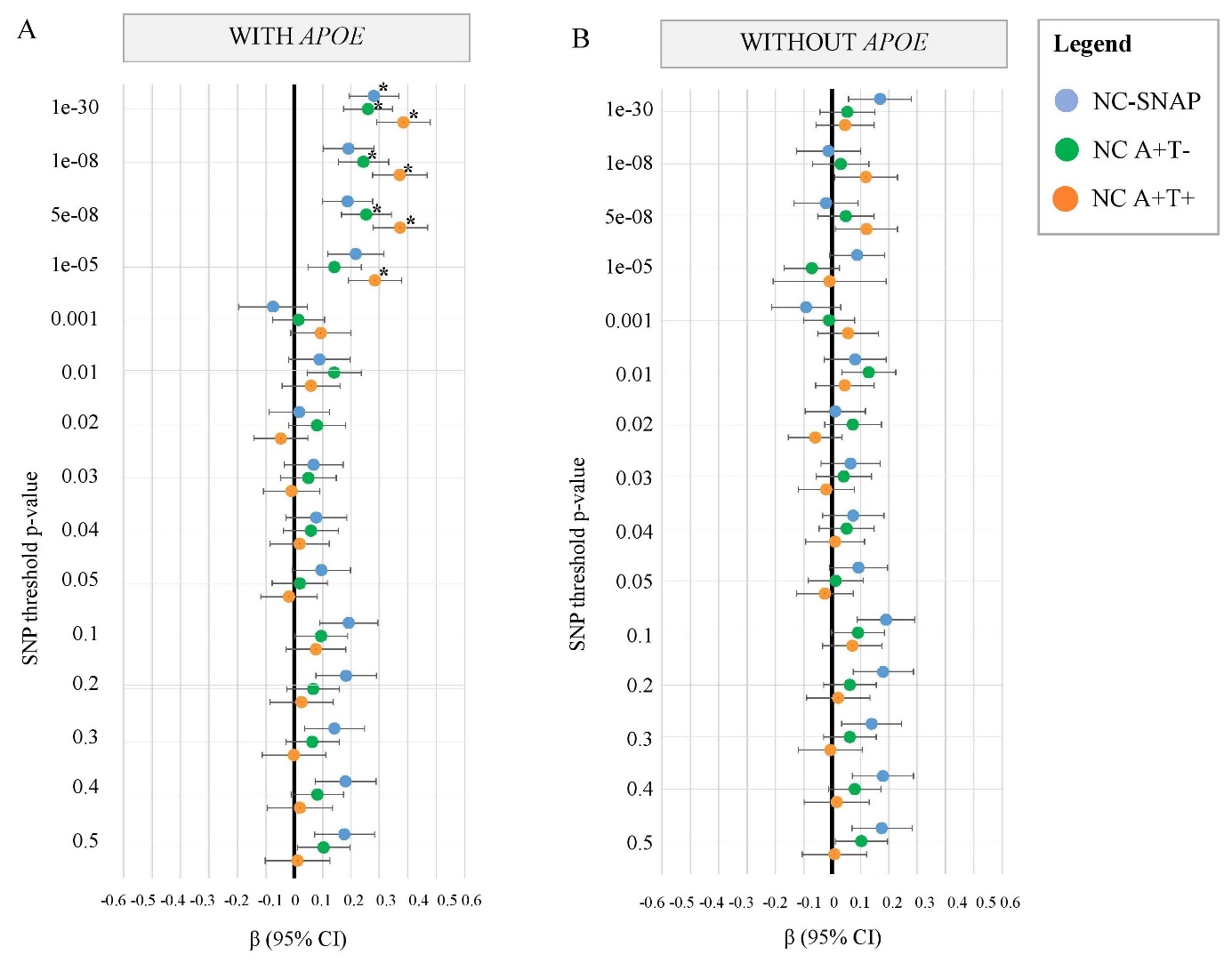


**Supplementary Figure 1. Polygenic risk score for Alzheimer’s disease (AD PGRS) across different single nucleotide polymorphism (SNP) p-value thresholds. (A)** AD PGRS with apolipoprotein E (APOE) region. Differences in AD PGRS (based on de Rojas et al.) for different SNP threshold P-values. The black line indicates AD PGRS of controls, the blue dots represent AD PGRS of NC-SNAP (A-T+), the green dots represent AD PGRS of NC A+T- and the orange dots represents AD PGRS of NC A+T+. AD PGRS were compared between groups using linear regression corrected for age and sex. **(B)** AD PGRS without APOE region. Differences in AD PGRS (based on de Rojas et al.) for different SNP threshold P-values. The black line indicates AD PGRS of controls, the blue dots represent AD PGRS of NC-SNAP (A-T+), the green dots represent AD PGRS of NC A+T- and the orange dots represents AD PGRS of NC A+T+. AD PGRS were compared between groups using linear regression corrected for age and sex. PGRS = polygenic risk score, AD = Alzheimer’s disease, SNP = single nucleotide polymorphism, NC = normal cognition, SNAP = Suspected non Alzheimer’s disease pathophysiology (A-T+), A+ = abnormal levels of CSF Aβ42, T+ = abnormal levels of CSF phosphorylated-tau.


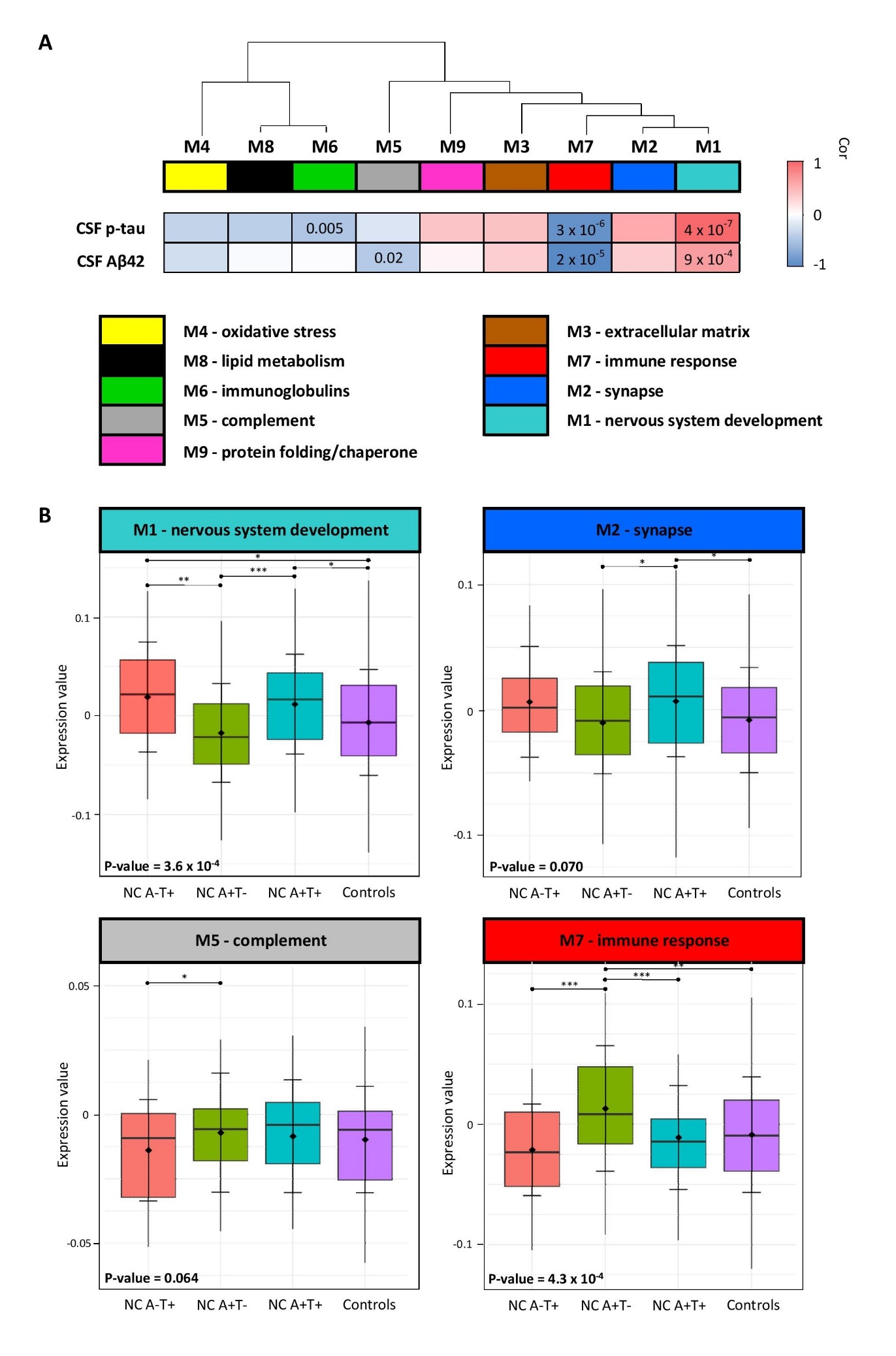


**Supplementary Figure 2. Protein Network Analysis. (A)** Protein correlation network consisting of 9 protein modules, each associated with specific pathways (bottom). The correlation of module eigenproteins with AD biomarkers (local CSF Aβ42 and p-tau Z-scores) was calculated using biweight midcorrelation. The strength of positive (red) and negative (blue) correlations is shown in a two-color heatmap, with p-values provided for all correlations with a p < 0.05. **(B)** Module eigenprotein levels by AT groups for each protein module that exhibited significant differences between groups. Differences in eigenprotein values between groups were assessed using the Kruskal–Wallis test, followed by the Wilcoxon test for pairwise comparisons. Significant differences are indicated by stars: *p < 0.05, **p < 0.01, ***p < 0.001. A+ = abnormal levels of CSF Aβ42, T+ = abnormal levels of CSF phosphorylated-tau, Aβ42 = amyloid β42, M = module, NC = normal cognition, p-tau = phosphorylated tau.

**Supplementary references**

1. van der Flier WM, Scheltens P, et al. Optimizing patient care and research: the Amsterdam Dementia Cohort. *J Alzheimers Dis.* 2014;41(1):313-327.

2. Somers C, Struyfs H, Goossens J, et al. A decade of cerebrospinal fluid biomarkers for Alzheimer's disease in Belgium. J Alzheimers Dis. 2016;54(1):383-395.

3. Visser PJ, Verhey F, Boada M, et al. Development of screening guidelines and clinical criteria for predementia Alzheimer's disease: the DESCRIPA Study. *Neuroepidemiology*. 2008;30(4):254-265.

4. Reijs BLR, Ramakers IHGB, Köhler S, et al. Relation of odor identification with Alzheimer's disease markers in cerebrospinal fluid and cognition. *J Alzheimers Dis*. 2017;60(3):1025-1034.

5. Estanga A, Ecay-Torres M, Ibañez A, et al. Beneficial effect of bilingualism on Alzheimer's disease CSF biomarkers and cognition. *Neurobiol Aging*. 2017;50:144-151.

6. Tautvydaite D, Kukreja D, Iceta S, et al. Interaction between personality traits and cerebrospinal fluid biomarkers of Alzheimer's disease pathology modulates cognitive performance. *Alzheimers Res Ther.* 2017;9(1):6.

7. Adamczuk K, De Weer A-S, Nelissen N, et al. Amyloid imaging in cognitively normal older adults: comparison between 18F-flutemetamol and 11C-Pittsburgh compound B. *Eur J Nucl Med Mol Imaging.* 2016;43(1):142-151.

8. Bos I, Vos SJB, Frisoni G, et al. Cerebrovascular and amyloid pathology in predementia stages: the relationship with neurodegeneration and cognitive decline. *Alzheimers Res Ther.* 2017;9(1):101.

9. Volluz KE, Schindler SE, Henson RL, et al. Correspondence of CSF biomarkers measured by Lumipulse assays with amyloid PET. *Alzheimers Dement.* 2021;17(S5):e051085.
